# Modeling the Hematopoietic Landscape

**DOI:** 10.3389/fcell.2019.00104

**Published:** 2019-06-18

**Authors:** Geoffrey Brown, Rhodri Ceredig

**Affiliations:** ^1^Institute of Clinical Sciences – Institute of Immunology and Immunotherapy, College of Medical and Dental Sciences, University of Birmingham, Birmingham, United Kingdom; ^2^National University of Ireland Galway, Galway, Ireland

**Keywords:** hematopoiesis, hematopoietic stem cells, fate determination, blood cells, immune cells

## Abstract

Some time ago, we proposed a continuum-like view of the lineages open to hematopoietic stem cells (HSCs); each HSC self-renews or chooses from the spectrum of all end-cell options and can then “merely” differentiate. Having selected a cell lineage, an individual HSC may still “step sideways” to an alternative, albeit closely related, fate: HSC and their progeny therefore remain versatile. The hematopoietic cytokines erythropoietin, granulocyte colony-stimulating factor, macrophage colony-stimulating factor, granulocyte/macrophage colony-stimulating factor and ligand for the fms-like tyrosine kinase 3 instruct cell lineage. Sub-populations of HSCs express each of the cytokine receptors that are positively auto-regulated upon cytokine binding. Many years ago, Waddington proposed that the epigenetic landscape played an important role in cell lineage choice. This landscape is dynamic and unstable especially regarding DNA methylation patterns across genomic DNA. This may underlie the receptor diversity of HSC and their decision-making.

## Introduction

Waddington (1957), developed the idea that the developmental fates of cells are somehow shaped by a continuous interplay between environmental influences and the actions of genes, which he dubbed “epigenetics”. He offered a celebrated pictorial depiction of this mysterious process (Waddington, 1957), in which embryonic cells and their progeny started their developmental journeys through his metaphorical landscape on the rolling uplands, and from there they coursed through a bifurcating series of steep-sided valleys, with little chance of sideways escape, and emerged as the various types of differentiated cells.

Early fate maps of hematopoiesis similarly envisaged branching development, from one initial hematopoietic stem cell (HSC) population, to the many types of differentiated blood and immune cells, with the progeny of HSCs progressing stepwise through several binary and irreversible fate choices until the various types of haematopoietic end-cells emerged. In this scenario, HSCs are cells that are able to either self-renew or commit to differentiation and thereby give rise to all of the blood and immune cell types. Haematopoietic progenitor cells (HPCs) are their progeny that have “chosen” to differentiate. They include cells that are multipotent and that have a sub-set of differentiation potentials, having undergone stepwise fate choices.

Since the late 1950s, “epigenetics” has since come to mean much more than Waddington’s original mid-20th century abstraction, and it now encompasses all of the many more recently discovered processes – such as DNA methylation and various modifications of histones – that modulate the functional outputs of genes without altering their underlying sequences.

Here we focus on how a HSC “choses” a particular pathway of development, leaving aside the substantial body of information on dormancy vs. self-renewal vs. differentiation *per se*. There has been similar progress in our views of the development of – and understanding of the complex relationships between – the various HSC-derived cell lineages, and we now recognize that the pathways to and functions of the multifarious hematopoietic cell types are less strictly demarcated than was initially thought. HSCs also give rise to many more end-cell types than we would hitherto have expected (Brown et al., 2018a). There has long been a tendency to view each kind of blood and/or immune cell as having a particular (set of) physiological role(s) and to define – and sometimes to sort – these cells based on a particular pattern of cell surface markers. However, this overlooks the degree to which many of the populations share some phenotypic and functional attributes. For example, B-lymphocytes, monocytes and dendritic cells are morphologically very different but are closely developmentally related (see below), and they all phagocytose and pinocytose foreign antigens, and process them.

Recently, the use of new tools have provided much more information about the characteristics of the individual cells that make up the HSC and HPC populations. Using the “index sorting” function of cell sorters, we can isolate single cells that have a precisely defined surface phenotype and then relate this to a variety of other characteristics – such as the genes the cell expresses and the proteins it contains. New technologies that include single cell mRNA sequencing and single-cell mass-spectrometry have allowed investigators to profile the genes expressed by a cell. Whilst these advances have allowed investigators to obtain much more information about the characteristics of an individual cell and a population of cells. The question of particular interest is how does a cell arrive at, and maintain, a particular phenotype.

The use of new approaches has revealed that HSCs and multipotent HPCs are heterogeneous regarding the proteins they express and their lineage predispositions. Knapp et al. (2018) characterized the most primitive CD34^+^ hematopoietic cells from human cord blood (CB). They analyzed the mass cytometry data obtained from individually assessed CD3^-^ CD34^+^ CD19^-^ CD11b^-^ CD38^-^ CD45RA^-^ CD90^+^ CD49f^+^ cells (CD49f^+^ CB) revealing extensive heterogeneity in the levels of the 40 proteins examined. Index-sorted CD49f^+^ CB were lentivirus barcoded and their lineage potentials examined by injecting pools of transduced cells into irradiated mice. The lineage content of clones varied widely. For 61 clones (at 30–38 weeks), a few (8%) yielded cells of many lineages, 13% yielded a B cell/granulocyte/macrophage mixture, 30% produced B cells, 36% gave rise to granulocytes and macrophages, and 13% only engrafted transiently. Optimization of the reconstitution assay to detect both lymphoid and myeloid outputs resulted in 8% of clones yielding cells of many lineages, 27% a myeloid/lymphoid mixture, 30% producing myeloid cells, 22% giving rise to lymphoid cells, and 14% engrafted transiently. A caveat to this heterogeneity of primitive CB cells is that they might be a mixture of HSCs and committed cells. Even so and as below, the cells that we best purify as HSCs, and as a particular downstream population of cells, are truly heterogeneous in various ways. Perhaps, this alleviates the need to delineate HSCs vs. HPCs (as above) and instead there is merely a population of blood cell precursors with various lineage affiliations and other properties.

Paul et al. (2015) similarly examined the transcriptional patterns of individual mouse bone marrow myeloid progenitors, and assigned cells to one of seven groups along a neutrophil/basophil/eosinophil/monocyte/dendritic cell/erythrocyte/megakaryocyte spectrum. They sub-classified their cells into 18 populations, with variable degrees of lineage affiliation. Cells in adjacent groups were more similar, whereas those at the extremes were quite distinct. We might therefore visualize the differences between cell types as a continuum (see later).

## Lineage-Affiliation Can Be Initiated as Early as Within HSC

For many years, we assumed that sorting of cell populations by means of the multiple surface markers designed to harvest either HSC or various multipotent HPCs would yield a population of cells that is both relatively homogeneous and mostly multipotent. We presumed that a large proportion of these cells had the potential – either when appropriately cultured or when engrafted into irradiated recipients – to give rise to a wide spectrum of hematopoietic cells. However, the recent availability of information on the characteristics and lineage outputs from individual HSCs/HPCs has led to a fundamental change in view. For example, in one of the experiments mentioned above a substantial proportion of the CD49^+^ CD34^+^ human CB cells harvested as “primitive HPCs” only gave rise to B lymphocytes in engrafted animals.

In keeping with lineage affiliation starting earlier than previously thought, even in HSCs, human adult bone marrow CD34^+^ cells are mainly cells with uni-potent myeloid or erythroid potential alongside some multipotent progenitors. There are few oligo-potent progenitor intermediates, though fetal liver contains HPCs with megakaryocyte/erythroid/myeloid and megakaryocyte/erythroid fates (Notta et al., 2016). If lineage-affiliation occurs as early as within HSC, then their progeny that we ring-fence by the use of surface markers and view as a multipotent cells should be a mixture of cells with each having a distinct lineage signature. Indeed, this is the case for a population of mouse cells first described as early progenitors with lymphoid and myeloid potential (EPLM) (Balciunaite et al., 2005a). A “primitive” sub-population of EPLM lacks expression of the markers Ly6D, SiglecH, and CD11c and RNA sequencing of single cells revealed that they really are a mixture of cells with either a myeloid, dendritic cell or lymphoid signature; few have lymphoid and myeloid potential (Alberti-Servera et al., 2017). For the Ly6D^-^ SiglecH^-^ CD11c^-^ population, lineage affiliations must have occurred at an earlier stage of development, perhaps within HSCs. However, Kauts et al. (2016) have argued that intermediate oligo-potent populations are important for the production of adult mouse blood cells and that the embryo produces blood cells without this need.

## A Pairwise and Continuum-Like Model for Hematopoiesis

**FIGURE 1:**
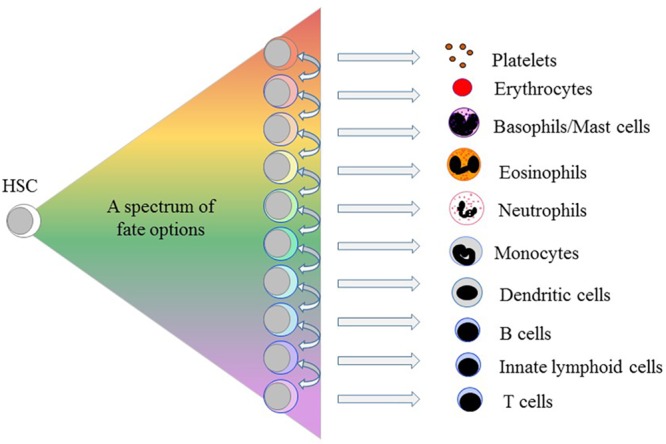
A spectrum of fate options is available to hematopoietic stem cells. By contrast to the progeny of HSCs moving stepwise, through serial fate decisions, to various cell-types each HSC chooses from a spectrum of all of the end cell options. The arrows indicate that for each lineage there are pairwise neighbors in a relatedness hierarchy. HSCs and their progeny remain versatile because having selected a cell lineage they may still “step sideways” to an alternative, albeit closely related, fate(s).

In 2009, we proposed a pairwise and continuum-like view of the lineages open to the HSCs. A lack of arrows on the diagram to show a preferred route (or routes) to each end-cell type reflected our viewpoint that several binary and irreversible choices do not underlie the development of end-cell types (Ceredig et al., 2009). Instead, each HSC self-renews or chooses from a spectrum of all of the end cell options – then can “merely” differentiate (Figure 1). Similarly, a schematic representation of the data obtained for single-cell proteome measurements for differentiating human CD34^+^ CB cells indicates a landscape topology with multiple directional paths emanating from the most primitive HSCs (Knapp et al., 2019). Whilst HSC can therefore affiliate directly to a single cell lineage, they and their progeny remain versatile. They may still “step sideways” to an alternative fate. Megakaryocyte-primed human HSCs can “step sideways” to erythropoiesis and because of a common dependence on the transcription factor (TF) GATA-1 there is a “shared” trajectory (Psaila and Mead, 2019). In this scenario and when cells deviate “sideways” from their chosen fate – their most probable route – they tend to switch most readily to a path that leads to their pairwise neighbors in a relatedness hierarchy (see Figure 1 and below). For example, an individual HSC committed to macrophage development is more likely to step sideways to generate neutrophils or dendritic cells than give rise to B-lymphocytes, though the degree of sideways -shuffling *in vivo* is unknown. As cells move toward terminal differentiation, the extent of change to lineage preference progressively narrows.

Initially, we placed the cell lineages next to one another from the options available to bi-potent cell populations, as revealed for bone marrow cells using semi-solid medium, and the sets of options available to mouse and human oligo-potent progenitors, as revealed in cell culture experiments (Brown et al., 2007; Ceredig et al., 2009). Additionally, a particular lineage ties to its pairwise partners regarding attributes they share early during development, for example, their use of TFs, and shared functional characteristics, as mentioned above. By definition, a continuum does not have precise boundaries in keeping with sharing and a gradual and continuous process of commitment to an end-cell type. Velten et al. (2017) arrived at very similar links between the cell lineages by constructing the developmental trajectories for human HPCs from integrating single-cell RNA sequence data with data from single cell cultures. They placed cell lineages in the order B cell, monocyte/dendritic cell, neutrophil, eosinophil/basophil/mast cell, megakaryocyte and erythrocyte (Velten et al., 2017) (see Figure 1), arguing that HSC lineage commitment is a continuous process.

HSCs and their progeny showing some degree of early lineage affiliation are nevertheless still versatile. The development of mouse progenitor thymocytes exemplifies cells that appear to have made a lineage “choice” but are still able to divert to a different end-cell fate. Double negative (DN) 1 and DN2 mouse progenitor thymocytes have begun to arrange their T cell receptor β genes and they progress through the later stages of T-cell developmental in fetal thymus organ cultures. They are on their way to becoming T cells but can still give rise to macrophages and natural killer cells (Porritt et al., 2004; Balciunaite et al., 2005b). For functional macrophages, DN1 and DN2 were cultured on ST-2 stromal cells and with interleukin (IL)-7. ST-2 cells produce a low level of macrophage colony-stimulating factor (M-CSF) and macrophage colonies from DN1 and DN2 thymocytes did not occur when investigators used the M-CSF-non-secreting OP9 stromal cell line for support. Culture of DN1 and DN2 cells on OP9 cells and in the presence of IL-7 and IL-2 led to functional natural killer cells but this was not to any large degree dependent on the presence of IL-7 (Balciunaite et al., 2005b). In both these instances, the presence of a particular cytokine, M-CSF and IL-2, was required to “divert” the lineage affiliation of progenitor thymocytes, indicating an importance of cytokines to lineage choice. We have argued that the range of cell types seen in the colonies formed by HPCs dispersed in semi-solid medium colonies reflect cells that are shuffling sideways because they are out of their normal social environment regarding fate restriction (Brown et al., 2018b).

Nestorowa et al. (2016) RNA sequenced more than 1600 single mouse HSCs and HPCs and then constructed expression maps to reveal the HSC trajectories along the erythroid, granulocytic/macrophage and lymphoid pathways. They proposed broad trajectories with cells having the option of moving to the left or right of a chosen developmental trajectory. Similarly, Olsson et al. (2016) observed bi-potential patterns of gene expression in their analysis of the lineage status of HPCs. For example, granulocyte/macrophage progenitors (GMP) express Gfi1 and Irf8 mRNAs at low levels and increase expression of these genes during neutrophil and macrophage specification, respectively. Olsson et al. (2016) argue that bi-lineage states and bursts of alternative gene expression are an important feature of cell-fate specification.

## Some Haematopoietic Cytokines Instruct Lineage Fate

Sub-populations of HSCs express cytokine receptors that associate with a particular cell lineage(s). Mouse HSCs express the receptor for macrophage colony-stimulating factor (M-CSFR, and termed CSF-1R and c-fms) at their cell surface (Kondo et al., 2000; Mossadegh-Keller et al., 2013), and in particular by 19% of LT-HSC and 23% of short-term reconstituting HSCs (ST-HSC) (Mooney et al., 2017). Thirteen percent and 19% of mouse LT-HSC and ST-HSC, respectively, express mRNA for the erythropoietin receptor (EpoR); the lack of an antibody at the time precluded the analysis of protein expression (Mooney et al., 2017). Human HSCs are enriched within CD34^+^CD38^-/dim^ bone marrow cells and some of these cells express the EpoR, as measured using biotinylated recombinant Epo and a streptavidin conjugate (Shinjo et al., 1997). The ligand for the fms-like tyrosine kinase 3 (Flt3L) is lympho/myeloid affiliated. The fms-like tyrosine kinase 3 (Flt3) is expressed by 5% of mouse LT-HSC and 8% of ST-HSC and post-treatment with Flt3L the downstream ribosomal protein S6 is phosphorylated. One percent of LT-HSCs and 3% of ST-HSCs co-express Flt3 and the M-CSFR and co-expression of the mRNAs encoding Flt3 and EpoR was rare (Mooney et al., 2017). Mobilization studies using chimeric mice reconstituted with cells from wild type and mice deficient in the receptor for granulocyte colony-stimulating factor (G-CSFR) have shown that transplantable HSCs express this receptor (Liu et al., 2000), and granulocyte-colony stimulating factor (G-CSF) increases the frequency of HSCs in bone marrow (Schuettpelz et al., 2014). Primitive mouse HSCs express low to moderate levels of the receptor for granulocyte/macrophage colony-stimulating factor (GM-CSF) (Kondo et al., 2000). A sub-set of human CD34^+^CD38^-/dim^ bone marrow cells expresses the thrombopoietin receptor (TpoR) (Ninos et al., 2006) and the platelet-associated von Willebrand factor and biased toward the platelet and myeloid pathways (Sanjuan-Pla et al., 2013). Thrombopoietin (Tpo) has a role in maintaining HSC (De Graaf and Metcalf, 2011) and is essential to maintain the TpoR^+^ sub-set.

**FIGURE 2:**
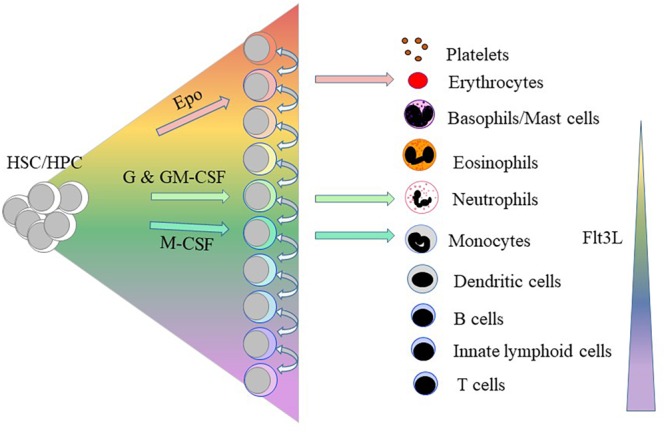
Some of the haematopoietic cytokines instruct the lineage fate of haematopoietic stem and progenitor cells. Erythropoietin (Epo), granulocyte colony-stimulating factor (GSF)/granulocyte/macrophage colony-stimulating factor (GM-CSF) and macrophage colony-stimulating factor (M-CSF) instruct hematopoietic stem cells (HSCs)/hematopoietic progenitor cells (HPCs) to adopt erythroid, neutrophil and macrophage fates, respectively. Above a certain threshold level, the ligand for the fms-like tyrosine kinase 3 (Flt3L) drives HSC development along the myeloid-lymphoid pathways as opposed to the generation of megakaryocyte and erythroid progenitors.

Erythropoietin (Epo) supports erythroid progenitor cells (Koury and Bondurant, 1990); G-CSF supports granulocyte precursors (Demetri and Griffin, 1991; Metcalf, 1993); M-CSF the development of macrophages and DCs (Hume and MacDonald, 2012) and GM-CSF precursors of granulocytes and macrophages (Gasson, 1991; Metcalf, 1993). For many years, we therefore viewed these cytokines as regulators of HPC survival and proliferation (Cross et al., 1997). We now know they are instrumental in determining HSC/HPC erythroid, granulocyte and macrophage fates (Figure 2; reviewed in Brown et al., 2018b). Epo commits multipotent mouse HPCs to the erythroid fate, initiating a program that includes the expression of the erythroid-affiliated TF GATA1 (Grover et al., 2014). In 1982, Metcalf and Burgess cultured each of the daughter cells of mouse granulocyte-macrophage colony forming cells in either GM-CSF or M-CSF leading to the generation of granulocytes and macrophages, respectively (Metcalf and Burgess, 1982). Continuous *in vivo* tracing of cells provided proof that cytokines can instruct a choice between the granulocyte and macrophage fates whereby Rieger et al. (2009) showed that G-CSF and M-CSF instruct mouse granulocyte/macrophage progenitors to adopt the granulocyte and macrophage pathways, respectively. Single cell studies have shown that M-CSF instructs myeloid lineage fate in mouse HSCs including expression of the TF PU.1, a regulator of myeloid development (Mossadegh-Keller et al., 2013).

The instructive action of Flt3L is concentration dependent. Mouse HSC lie within populations of bone marrow cells lacking expression of cell lineage markers (Lin^-^) and that express the Sca-1 (Ly6 family) antigen and the receptor for the stem cell factor CD117 or c-kit; and termed LSK. Exposure of LSKs to Flt3L above a certain threshold level drives their development along the myeloid-lymphoid pathways and suppresses the generation of megakaryocyte and erythroid progenitors (Tsapogas et al., 2014). Similarly, over-expression of Flt3 in megakaryocyte/erythroid progenitors led to these cells differentiating toward granulocytes and macrophages, and upregulation of PU.1 (Onai et al., 2006).

An instructive vs. a permissive action of Flt3L on HPCs is also highly cell type dependent. CLPs and EPLMs are downstream of LSKs and Flt3L plays a permissive role during the development of these cells along the B cell lineage (von Muenchow et al., 2016). In other words, FLt3L is essential for the survival and proliferation of cells that are already committed to the B cell lineage.

Concentration and cell context are both important for cytokine action and therefore survival/proliferation vs. instructive actions of cytokines are not binary alternatives *per se*. Low levels of multiple cytokines during steady state conditions are sufficient to sustain cell survival/proliferation and the steady-state production of blood cells in normal ratios. By contrast, studies showing cytokines to be instructive have exposed cells to a much higher level, as required for the instructive action of FLt3L. Cytokine levels increase substantially locally and systemically, for example, during an infection, which might influence the lineage output of HSCs/HPCs by instructing lineage fate. The hematopoietic and immune system therefore respond to an emergency in the most appropriate manner.

As to filling in the gaps on identifying instructive cytokines, there are at least 33 cytokines and over 100 genes that encode cytokine-like activities (Dinarello, 2007). The emergence of adaptive immunity has been a major driver to the evolution of this substantial array of cytokines because their evolution parallels that of the immune system (Liongue et al., 2016).

## Sharing of Intracellular Signaling Pathways

It seems unlikely that a unique intracellular signal(s) generated by an instructive cytokine receptor encrypts specificity to the choice of pathway. Replacement of the cytoplasmic and signaling domain of the G-CSF receptor with the EpoR signaling domain and expression of this chimeric receptor in homozygous mice did not specify signals to skew hematopoiesis toward erythropoiesis (Semerad et al., 1999). On the contrary, the activation of different transduction pathways supports erythropoiesis (reviewed in Constantinescu et al., 1999), including the phosphoinositol-3 kinase (PI3-K)/Akt, Erk/MAPK and JAK–STAT pathways, and protein tyrosine phosphatases (Richmond et al., 2005). Signaling by the PI3-K/mitogen-activated kinase (MAP) pathway is sufficient, but is not essential, for erythroid differentiation because a mutant EpoR lacking the phosphorylated tyrosine that recruits the p85 subunit of PI3-K kinase, leading to activation of the MAP kinase (ERK2) pathway, adequately supports erythroid differentiation. Furthermore, mutant EpoRs that active only some of the proteins support erythropoiesis when expressed in EpoR^-/-^ progenitors. An argument in favor of specificity within signal transduction is that Epo up-regulation of pre-erythroid gene expression within LSKFlt3^-^CD150^+^ HSCs is dependent on PI3-K activation whereas this is not the case for pre-myeloid gene expression which was unaffected by PI3-K inhibition (Grover et al., 2014).

**FIGURE 3:**
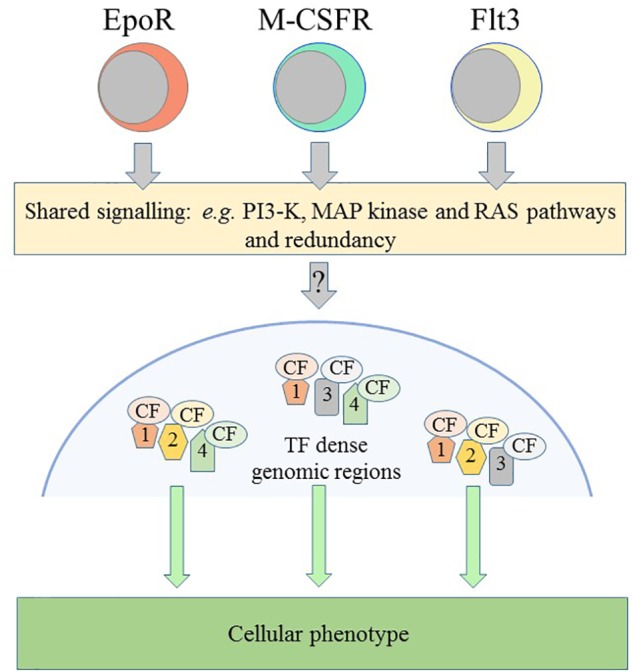
Schematic representation of some of the controls on cellular phenotype. The receptors for erythropoietin (Epo) and for macrophage-colony stimulation factor (M-CSFR) and the fms-like tyrosine kinase 3 (Flt3) can instruct erythroid, macrophage and lymphoid/myeloid fates, respectively. Receptors share the use of signaling pathways and there is redundancy in their use. Various transcription factors, the colored shapes labeled 1, 2, 3, and 4, and their cofactors, the colored CF ovals, target and densely occupy certain genomic regions that are important for the specification of a cellular phenotype. PI3-K, phosphatidylinositol 3-kinase; MAP, mitogen-activated kinase.

There is shared usage of a “core” signaling network by the cytokine receptor family, as for example activation of MAP kinase by EpoR (Richmond et al., 2005), M-CSFR (Curry et al., 2008) and Flt3 (Hayakawa et al., 2000; Figure 3). This is not too surprising because the outputs must converge on controlling cell survival and proliferation. However, binding of a cytokine to its receptor leads to the increased expression of a lineage-affiliated TF that reinforces receptor expression, as follows.

## Auto-Regulation of Cytokine Receptors

For many years, investigators have argued that the action of a constellation of TFs across the genome underlies the unique and shared usage of gene cassettes to specify a cell’s fate (Figure 3). More than 50 TFs influence the nature of HSCs and TFs and their cofactors are a densely connected regulatory network as required to specify a cell’s phenotype (reviewed in Wilson et al., 2011). Where TFs and cofactors reside within cells is important, for example, EpoR signaling leads to proteins translocating from the cytoplasm to the nucleus to form DNA-binding complexes that recognize the c-fos promotor (Finbloom et al., 1994).

Are there pivotal and master regulators of lineage specification? As revealed by the use of PU.1-GFP mice, M-CSF stimulated expression of PU.1 in some LT-HSC leads to myeloid lineage specification (Mossadegh-Keller et al., 2013). Similarly, PU.1 and GATA-1 activation mark the specification of HSCs into myeloid/lymphoid vs. myeloid/erythroid lineages, respectively (Arinobu et al., 2007). Dahl et al. (2003) have argued that the level of PU.1 is important with the need for a higher level for macrophage than for neutrophil development. However, Hoppe et al. (2016) have shown that the protein ratios of the myeloid/lymphoid-affiliated PU.1 and erythroid-affiliated GATA-1 do not direct lineage choice, and merely reinforce pre-existing myeloid and erythroid fates, respectively. The finding that PU.1 levels change after myeloid lineage choice also refute a PU.1/GATA-1 switch (Strasser et al., 2018). The shared rather than the unique usage of TFs argues also against any particular TF providing a “master” signal. However, this is commensurate with placing cell lineages next to one another in the continuum model (Figure 2) whereby TFs can promote the development of adjacent cell types and suppress the development of a cell lineage that lies on either side of a fate (Ceredig et al., 2009).

**FIGURE 4:**
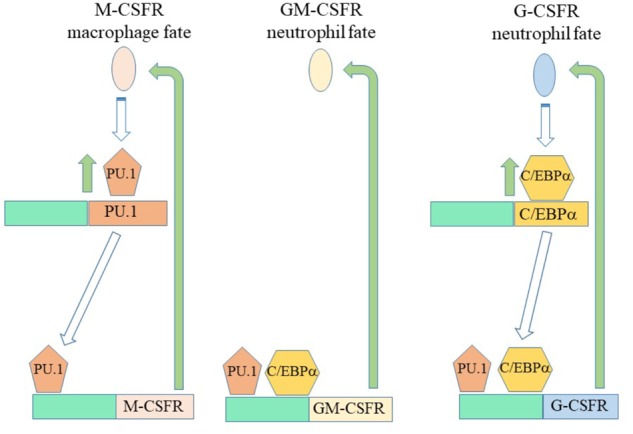
Auto and promiscuous regulation of the expression of cytokin receptors. Macrophage-colony stimulating factor and granulocyte/ macrophage-colony stimulating factor/granulocyte-colony stimulating factor can instruct macrophage and neutrophil fates, respectively. Macrophage-colony stimulating factor receptor (M-CSFR) instruction of HSCs leads to expression of the transcription factor PU.1. PU.1 in turn regulates expression of the M-CSFR, the granulocyte/macrophage-colony stimulating factor receptor (GM-CSFR) and the granulocyte-colony stimulating factor receptor (G-CSFR). G-CSF receptor signaling increases the expression of C/EBPα that regulates the expression of the GM-CSFR and the G-CSFR. A complex interplay between transcription factors and the expression of cytokine receptors might underlie meta-stable bi-lineage cell states and allow HSCs that express the M-CSFR and cytokine stimulated to co-express G-CSFR “side step” to an adjacent pathway (Figure 1).

A feature of some of the cytokine receptors that we know to be instructive is that their expression is positively auto-regulated (Figure 4). M-CSF treatment of mouse HSCs, which include cells that express M-CSFR, leads to the expression of PU.1 and genes for myeloid development. PU.1 also activates the M-CSFR promotor to auto-regulate increased expression of this receptor (Brach et al., 1991; Zhang et al., 1994; Stanley and Chitu, 2014). Additionally, PU.1 controls expression of the GM-CSFR, for neutrophil fate, and the G-CSFR (Smith et al., 1996) that can instruct neutrophil fate. Another regulator of G-CSFR is the CCAAT/enhancer binding protein (C/EBP) α - a neutrophil-affiliated TF whereby loss of C/EBPα leads to loss of myeloid programming (Hohaus et al., 1995; Smith et al., 1996; Bereshchenko et al., 2009). Signaling from the G-CSF receptor increases the expression of C/EBPα (Dahl et al., 2003) that supports neutrophil development. Like the M-CSFR, the G-CSFR is auto-regulated by C/EBPα that also controls the expression of the GM-CSFR that can also instruct neutrophil fate. Activation of Flt3 expression needs cooperation between MYB and C/EBPα (Volpe et al., 2015). Ligand-activation of Flt3 induces expression of both C/EBPα and PU.1 (Mizuki et al., 2003) whereby Flt3 expression might be auto-regulated by C/EBPα, expression of G-CSFR influenced by C/EBPα, and expression of M-CSFR and GM-CSFR influenced by PU.1. This complex interplay of cytokine receptors and TFs might underpin the meta-stable bi-lineage states favored by Olsson et al. (2016) and the near-neighbor arrangement of cell lineages in the pairwise model (Figure 1). M-CSFR^+^ HSCs stimulated by M-CSF might readily express G-CSFR to allow “side-ways” diversion to the adjacent neutrophil pathway (Figure 1), and vice versa for G-CSFR^+^ HSCs.

Does auto-regulation of gene expression play a more general role in a cell’s capacity to change and/or establish its’ nature. The most active metabolite of vitamin D 1α,25-dihydroxivitamin D_3_ binds to the vitamin D receptor, a TF, to drive the differentiation of many types of cells. In mouse HSC-like cells, and many other cell types, increased expression of the vitamin D receptor is auto-regulated by its ligand (Janik et al., 2017). Every cell, with the exception of erythrocytes, can produce a cytokine as well as respond to them, and some cytokines function as a non-released membrane protein (Dinarello, 2007). Human haematopoietic cell lines representing early myeloid cells co-express Flt3 and Flt3L, allowing self-determination of receptor activation to possibly drive differentiation (Brasel et al., 1995). A cytokine-mediated autocrine loop can also control activation of the M-CSFR (Menke et al., 2012), the G-CSF (Mueller et al., 1999) and the GM-CSFR (Rogers et al., 1994). Auto-regulation might therefore underlie the established expression of some lineage-affiliated genes as seen in global analyses.

## The Epigenetic Landscape and Genomic Variability

Various TFs target and densely occupy specific genomic regions and sites poorly occupied by TFs contain genes expressed at low level or absent expression (Figure 3; Chen et al., 2008). A different viewpoint is that cells might compartmentalize regions of chromatin with a particular degree of intrinsic noise. Chromatin re-modeling is central to both these possibilities and TFs play a role in this process. PU.1 has a PEST domain involved in protein-protein interactions and DNA methytransferases are among the many different partners (van Riel and Rosenbauer, 2014). PU.1 forms a complex with the *de novo* DNA methyltransferases Dnmt3a and Dnmt3b (Suzuki et al., 2006) and the PU.1-DNMT3b complex exists as monocytes differentiate to osteoclasts (de la Rica et al., 2013). We view histone tail acetylation as a means of opening chromatin. PU.1 binds the histone acetyltransferases CREB-binding protein and p300 (Yamamoto et al., 1999; Bai et al., 2005) and a histone deacetylase complex consisting of histone deacetylase 1 and mammalian Sin3a (Kihara-Negishi et al., 2001).

The landscape of the methylation status of regions of DNA parallels the specific attributes of cells as revealed by sorting primitive CD49f^+^ CB cells into six sub-populations, based on their relative expressions of surface CD33, CD45, and CD202b (TIE2): CD202b expression is a marker of long-term repopulating mouse HSCs (LT-HSC). The CD33^+^CD90^+^ sub-set contains all of the serial repopulating activity, as measured by transfer into sub-lethally irradiated mice, and these cells are also quiescent. Analysis of the DNA methylation profiles of single index-sorted CD49f^+^ CB cells had revealed differential methylation of regions of DNA regarding cell sub-sets and the CD33^+^CD90^+^ CB cells have a distinct profile (Knapp et al., 2018). They show a greater consistency in the methylation status of DNA regions than any other sub-set. Importantly, there was a significant relationship between differentially methylated regions and the transcriptional signatures of the CD33^+^CD90^+^ CB cells.

Histone modifications are important for fate specification as follows. The chromatin of human CD34^+^ HPC is devoid of the repressive histone mark H3K27me3 just after cell division. Treatment of these cells with the instructive cytokines G-CSF/M-CSF and Epo leads to the recruitment of the myeloid-affiliated C/EBPα and lympho/myeloid-affiliated PU.1 vs. the erythroid-affiliated GATA-1 to DNA. These TFs recruited to DNA just after replication and blocking replication and increasing H3K27me3 levels suppressed cytokine driven differentiation (Petruk et al., 2017). Moreover, Roy and Sridaran have used the pattern of histone modifications to identify mouse cell types and their relationship to one another. They examined the presence of enhancer-enriched (H3K27ac, H3K4me1, and H3K4me2) and promotor-enriched (H3K4me3) chromatin marks for 15 types of haematopoietic cells. They used a clustering approach to identify chromatin modules as a set of gene loci with the same activating and repressive histone modifications. From modules that were more or less similar between cells, they delineated closely and distantly related cell types. Each group of the following cell types - immature erythroid cells/mature erythroid cells, granulocyte-macrophage progenitors/ macrophages/monocytes and CD4/CD8 T lymphocytes - has marks that are more similar to one another that to any other cell types (Roy and Sridharan, 2017).

Non-coding micro-RNAs (miRNAs) and long non-coding RNAs (lncRNAs) are a further layer of epigenetic regulation and include both enhancer and antisense RNAs. A substantial amount of work deals with the role of non-coding RNAs in orchestrating HSC differentiation, and the differentiation of other cell types, that is outside the scope of this review. They are important to modulating the behavior of cells because they can interact simultaneously with many targets to alter the expression of various components of TF networks and signaling pathways. Petriv et al. (2010) profiled miRNAs regarding the developmental hierarchy of mouse haematopoietic cells and used the information to infer the relationships between cell lineages and the functional similarities of cells. Are there non-coding RNAs that guide lineage fate? LncRNAs play a role in the commitment of osteoblasts to adipocyte differentiation (reviewed in Yoshioka and Yoshiko, 2017) and non-coding RNAs are therefore possible triggers of cell fate.

Importantly, the epigenome is highly dynamic and unstable. In the case of human pluripotent stem cells, there is instability and variation in the DNA methylation of a subset of developmental genes and a postulate is that this confers a growth/survival advantage (Lund et al., 2012). DNA methylation patterns across genomic DNA are unstable because for sub-cloned normal fibroblast cells they are highly variable which is attributable to a low fidelity of inheritance of DNA methylation (Xie et al., 2011). Genomic instabilities in genes that are associated with development are important to evolutionary changes (Zhao et al., 2010) and an argument in favor of instability playing a role to diversify HSCs is one of ontogeny repeating phylogeny. Randomness of the epigenetic code, via stochastic methylation changes (Laird et al., 2004; Riggs and Xiong, 2004), might allow HSCs/HPCs to establish inter-individual variation in lineage options (as above), providing the basis for the openness of lineage options and cell decision-making.

## “Choosing” a Fate and Survivability

**FIGURE 5:**
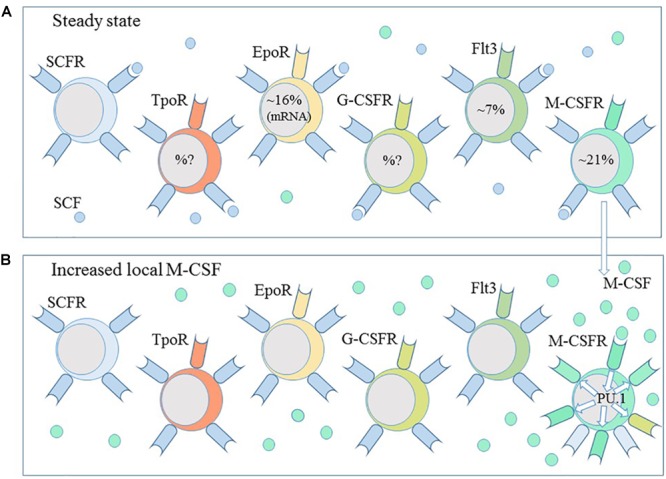
Haematopoietic stem and progenitors are a heterogeneous population of cells. **(A)** Subpopulations of haematopoietic stem and progenitor cells express the receptors for the cytokines thrombopoietin (TpoR), erythropoietin (EpoR), granulocyte-colony stimulating factor (G-CSFR) and macrophage-colony stimulating factor (M-CSFR) and the fms-like tyrosine kinase receptor (Flt3). A combination of stem cell factor (SCF), thrombopoietin and the ligand for Flt3 is sufficient to ensure the survival of HSCs *ex vivo*. **(B)** M-CSF instructs myeloid lineage fate in HSCs and the expression of PU.1. PU.1 regulates the expression of M-CSFR and G-CSFR. A local increase in M-CSF and autoregulation of M-CSFR expression might guide HSC toward a macrophage progenitor profile.

Whilst it is reasonable to accept that some cytokines drive lineage choice, cells must presumable express the gene encoding the receptor independently of the presence of the cytokine. Two aspects to this matter are how and why does this happen. As to how, a 1990s view of hematopoiesis focussed on the promiscuous and low-level expression of genes by HPCs. Expression of genes that are characteristic of different lineages led to the so-called “priming” of various developmental pathways (Cross and Enver, 1997; Enver and Greaves, 1998). We do not understand what controls this promiscuity and particularly its level. However, the genes primed in early human CD34^+^CD38^-^ HPCs included *SCL*, which regulates expression of the receptor for stem cell factor (SCFR) (Lécuyer et al., 2002), and *PU.1* (Cross and Enver, 1997), which regulates the promotors of *M-CSFR, G-CSFR*, and *GM-CSFR*. Early studies of single CD34^+^, lineage^-^ murine bone marrow cells observed co-expression of mRNA for the SCFR with that for the M-CSFR, G-CSFR, GM-CSFR, or EpoR (Hu et al., 1997). We might view HSCs/HPCs as one-and-the-same and a mosaic of cells regarding their expression of the SCFR, for survival as HSCs, together with one or more of the other cytokine receptors (Figure 5).

The extent to which the nature of the molecular structure of chromatin might underpin the promiscuity of HSCs is of particular interest. Chromatin domains that have a bivalent structure, termed bivalent chromatin domains, are important to the lineage choices that are available to cells, with a suggested role in maintaining pluripotency. These non-coding elements comprise of regions H3 lysine 27 methylation within which are smaller regions of H3 lysine 4 methylation. They coincide within regions of the genome that encode low-level expression of TFs that play key a role in development. A proposal is that the domains silence genes in embryonic stem cells but they are still “poised” and readily activated (Bernstein et al., 2006).

Raff postulated that cytokine, and other social, controls on cell survival select the fittest cells (Raff, 1992). Similarly, the basis of many homeostatic processes that govern the contribution of a cell type to a tissue/organism is competition between cells for resource including survival factors and cell-cell interaction (Johnson, 2009). The endeavor to survive applies to both developing and mature cells. Around 50% of oligodendrocytes in the developing rat optic nerve normally die, presumably due to competition for limiting amounts of survival signals (Barres et al., 1992). B lymphocyte survival in the periphery relates to the number of competitors and the availability of a survival factor(s) (McLean et al., 1997; Agenès, 2003).

As to why HSCs express lineage affiliated cytokine receptors may relate to the fact that the cytokines primarily ensure cell survival. A possible benefit of HSC individuality regarding the co-expression of cytokine receptors is to enhance survivability in a sporadically changing cytokine environment and/or competition for residency in a niche. Investigators mostly use stem cell factor (SCF), Tpo and Flt3 to ensure HSC survival in culture (De Graaf and Metcalf, 2011), including the HSCs expressing the M-CSFR^+^ (Mooney et al., 2017). However, SCF might become scarce *in vivo* and M-CSF, as available, signal to a SCFR^+^ M-CSFR^+^ HSC to up-regulate the low level of M-CSFR^+^ expression. Auto-regulation of this receptor ensures a cell “knows” which receptor to up-regulate, to optimize a “winner” survival status. The advantage of this is that cells can switch back and forth between the two survival modes. Additionally, we might expect a substantial local increase in the level of M-CSF to guide M-CSFR^+^ HSC toward macrophages that are then adapted and addicted to M-CSF for survival, including M-CSF signaling control of glucose uptake (Chang et al., 2009). In keeping with this cytokine-enforced selection, HSCs respond to chronic and sustained erythroid stress, and sustained exposure to high Epo levels *in vivo*, by displaying an erythroid progenitor profile, thereby bypassing other options to replenish erythrocytes (Singh et al., 2018).

How does choice arise in the first place regarding priming/expression of, for example, either the M-CSFR or the EpoR? Chromatin remodeling is indispensable for normal hematopoiesis in mice and the development of both the myeloid and lymphoid cell lineages (Han et al., 2019). The nascent status of chromatin is important because there is engraving of chromatin, including demethylation of CpG dinucleotides at critical TF binding sites, prior to cell lineage specification (Tagoh et al., 2004). What then drives the pattern of this landscape to specify and/or sustain the choice of a lineage? As considered above, perhaps inherent instability/lack of fidelity in the DNA methylation pattern across genomic DNA and/or chromatin activating and repressive histone modifications leads to the generation of a pool of HSCs with individual lineage biases as to a cytokine receptor(s) and a survival “winner” status.

## Implications for Leukemia

Whilst lineage affiliation can occur within HSCs, they nevertheless remain versatile. Many leukemias and cancers arise in a single stem cell and the malignant cells are forced mostly down one developmental pathway. Sánchez-Garcia and colleagues have argued that restriction of a leukemic HSC to one pathway occurs by oncogenes orchestrating the epigenome toward a leukemic cell lineage – with additional oncogene insults then converting this cell into a leukemia stem cell (LSC). Activity of the first oncogene is neither necessary to maintain LSCs nor for disease progression (Brown and Sanchez-Garcia, 2015; García-Ramírez et al., 2018; Vicente-Dueñas et al., 2018; Vicente-Dueñas et al., 2019). Its role is to focus HSC options into only one pathway, thus restricting LSCs and their progeny to that pathway. In this model, oncogene-restriction of the spectrum of options available to HSCs to just one pathway/fate, in other words a loss of versatility, is central to the initiation of leukemia. The oncogenic fusion proteins are important. For example, the BCR-ABL and BCR-ABLp210 oncogenes have roles in human leukemia and initiate leukemia when the expression of each oncogene is restricted to HSCs in transgenic mice. Each mouse line developed B-lymphocyte and chronic myeloid leukemia, respectively, typifying the human diseases (Brown and Sanchez-Garcia, 2015; García-Ramírez et al., 2018).

The instructive action of cytokines and TF-mediated auto-regulation of their receptors have implications to our understanding of leukemia. PU.1 plays a role in the perturbation in differentiation seen in acute myeloid leukemia (AML). Heterozygous mutations of the *PU.1* gene are found in some AML patients and 7 of the 9 mutations identified in 126 patients led to a deficiency in PU.1 binding to and transactivation of the M-CSFR promotor (Mueller et al., 2002). Mice with *PU.1* alleles that reduce expression to 20% of the normal level accumulate abnormal precursors that preserve responsiveness to G-CSF, for survival and important to neutrophil fate, whereas responsiveness to M-CSF and GM-CSF, for monocyte vs. neutrophil fate, is disrupted (Rosenbauer et al., 2004). The link between PU.1 and epigenetic control is pertinent to *PU.1* gene-mediated perturbations in AML (see above) whereby re-setting or switching-off epigenetic processes might provide an approach to normalizing leukemia cells.

Mutations in the *CEBP*α gene occur in roughly 10% of AML patients (Pabst and Mueller, 2009), but there is confusion about whether CEBPα expression is low or high in patients’ cells (Pabst and Mueller, 2009; Gholami et al., 2019). Epigenetic alterations of C/EBPα are a frequent event and epigenetic treatments can lead to down-regulation of this TF (Hackanson et al., 2008). Myb regulates Flt3 expression. One of the most frequent translocations observed in AML is t(9;11) and the *Myb* gene is up-regulated in cells from these patients (Lee et al., 2006) and over-expression of Myb contributes to leukemogenesis in human AML (K562)-SCID chimeric mice (Ratajczak et al., 1992). Relatively small changes to the levels of these TFs might “set” the lineage fate of a LSC via a profound change to the level of auto-regulated expression of a particular cytokine receptor(s).

## Perspectives and Conclusion

A cytokine-mediated instructive vs. a stochastic process, whereby the cytokines are merely permissive for differentiation, is far too simple a viewpoint of decision-making by HSCs/HPCs. It is reasonable to conclude that both nature, the innate attributes of cells, and nurture, by cytokines, play a role in specifying the lineage fate of a cell. Epo, G-CSF, M-CSF, GM-CSF and Flt3L can instruct cell lineage. Their receptors share use of the known intracellular signaling pathways, but signaling does lead to the expression of an appropriate lineage-affiliated TF(s). There is little evidence for a “master” TF regulator, or mix, for each of the individual cell lineages. However, the clustering of a variety of TFs to chromosome regions is important and innate instability to this landscape would endow individuality within HSCs, including perhaps the variable expression of cytokine receptors. In turn, receptors sensing a change in the environment as to the presence or absence of a cytokine would “guide” a cell toward a new identity. To commit to/arrive at a new identity, there is regulation of distant cassettes of genes and genome-wide mapping of TF binding events is feasible, but this is descriptive. From all of the above, a prediction is that the capture of chromatin signatures regarding regulatory elements that lie outside the boundaries of a gene locus coupled with information about the lineage intent of a cell population will advance our understanding of decision-making by HSCs.

## Author Contributions

All authors listed have made a substantial, direct and intellectual contribution to the work, and approved it for publication.

## Conflict of Interest Statement

The authors declare that the research was conducted in the absence of any commercial or financial relationships that could be construed as a potential conflict of interest.
